# Properties of Rennet Cheese Made from Whole and Skimmed Summer and Winter Milk on a Traditional Polish Dairy Farm

**DOI:** 10.3390/ani10101794

**Published:** 2020-10-02

**Authors:** Grażyna Czyżak-Runowska, Jacek Antoni Wójtowski, Danuta Gogół, Janusz Wojtczak, Ewa Skrzypczak, Daniel Stanisławski

**Affiliations:** 1 Department of Animal Breeding and Product Quality Assesment, Faculty of Veterinary Medicine and Animal Science, Poznan University of Life Science, Złotniki, ul. Słoneczna 1, 62-002 Suchy Las, Poland; grazyna.czyzak-runowska@up.poznan.pl (G.C.-R.); puchaladanuta@gmail.com (D.G.); janusz.wojtczak@up.poznan.pl (J.W.); ewa.skrzypczak@up.poznan.pl (E.S.); 2 Computer Lab, Poznań University of Life Sciences, Wołynska 33, 60-637 Poznan, Poland; daniel.stanislawski@up.poznan.pl

**Keywords:** small family farms, season, color, hygienic quality, texture

## Abstract

**Simple Summary:**

Milk from traditional family farms is a valuable raw material for cheese making. The aim of the study was to compare the textural and physicochemical characteristics, as well as the organoleptic properties, of soft rennet cheese from the milk of Polish Holstein–Friesian cows. The tests were carried out on 24 cheeses made from the bulk milk in the two production seasons: summer (July–September) and winter (January–March). The results indicate that both the season and the fat content of the milk affected the physicochemical (acidity, color) and rheological parameters (firmness and stickiness) of the cheese. What is more, the fat content of the milk had a more significant effect on the organoleptic parameters of the cheese than the season. In addition, low-fat cheeses received satisfactory organoleptic assessments, which indicates that they can serve as substitutes for full-fat cheeses for people looking for low-fat products.

**Abstract:**

The aim of this study was to compare the rheological and physicochemical parameters, as well as the organoleptic properties, of soft rennet cheese made from whole and skimmed milk in different seasons on a traditional family farm. We analyzed milk from twenty Polish Holstein–Friesian cows for basic composition, number of somatic cells, acidity, and color in terms of the Comission Internationale de l’Eclairage (CIE) lightness*redness*yellowness (L*a*b*) system, and 24 cheeses in terms of texture, acidity, color in terms of the CIE L*a*b* system, and organoleptic parameters in summer and winter. We determined the effects of the season and the fat content of milk on the pH, titratable acidity, color, firmness, and stickiness of the cheese. Cheeses from summer milk showed greater acidification than those from winter milk (*p* ≤ 0.05). Skimmed milk cheeses from both seasons showed increased firmness and stickiness, and worse organoleptic characteristics, particularly in taste and consistency, than whole milk cheeses (*p* ≤ 0.05). The highest level of yellow (b*) was found in whole milk summer cheeses; those produced in winter were 16% less yellow. Milk from traditional family farms is a valuable raw ingredient for the production of soft, unripe rennet cheese. However, the variability of organoleptic characteristics related to the season should be taken into account in cheese production. Skimmed cheese can serve as an alternative to full-fat cheese, especially for people looking for low-fat products, regardless of the time of year.

## 1. Introduction

Cows are the most numerous species of dairy animals, accounting for as much as 83% of global milk production [1]. Milk from traditional family farms, where cows graze on pastures, is of particular interest to consumers. This milk is a valuable raw ingredient in the production of cottage cheese, rennet cheese, fermented milk drinks, cream, and butter [2]. Its great nutritional value should be emphasized, especially due to its high levels of bioactive substances, resulting from traditional forms of animal nutrition [3,4,5,6,7,8]. In addition, traditional products are an important source of income for farms, and are sought after by consumers. Their production also contributes to environmental protection [9,10]. In addition, among those seeking a healthy lifestyle, there is a trend of consuming natural products, including cheeses with reduced calorie content. Reduced-fat food products can help prevent obesity and other diet-related diseases. However, the removal of fat often impairs the sensory and textural properties of foods, leading to low consumer acceptance [11]. Fat is the major substance defining milk’s energy value and makes a major contribution to the nutritional properties of milk [12]. Moreover, milk is a valuable source of fat-soluble vitamins, which are important for consumer health. Their content per 100 g of cow’s milk is relatively high and amounts to 126 International Units (IU) (vitamin A), 2 IU (vitamin D) and 0.1 mg (vitamin E) [12], which should be taken into account when consuming skimmed milk dairy products.

Most of the research on cheeses from traditional farms concerns ripened cheeses [13,14], and there have been few research papers on the properties of soft, unripe cheeses intended for immediate consumption. Due to the traditional animal feeding systems used in summer (pasture) and winter (silage and root crops) on small and medium-sized Polish farms, the season in which the milk is collected can alter the characteristics of the cheese produced from it. When analyzing dairy products manufactured using milk from a commercial dairy herd (UK), Chen et al. [15] demonstrated that the season does not have a significant impact on the properties of soft cheese (apart from its hardness), which can therefore be produced throughout the entire year.

The aim of the study was to compare the textural and physicochemical characteristics, as well as the organoleptic properties, of soft rennet cheese made from whole and skimmed milk collected in summer and winter.

## 2. Materials and Methods

### 2.1. Farm Characteristics

The experiment was performed on a traditional Polish dairy farm near Poznań (west of Poland, 52°24′ N, 16°55′ W; 69 meters above sea level (m.a.s.l.), during the summer and winter of 2018 and 2019. The area of the farm is 37 ha, of which 14 ha is arable land intended for sowing cereals, and 23 ha meadows and pasture. An additional 17 ha of meadows are leased out. Poor soils predominate on the farm. Cows in the herd undergo milk quality monitoring. Heifers for replenishing the herd are bred on the same farm.

### 2.2. Diet and Feeding

The dairy cows on the farm are fed three times a day in winter and twice in summer. In the autumn–winter season (from October to April), the basic volume ration consists of maize silage (17 kg fresh weight), hay silage (11 kg), ensiled beet pulp (15 kg), and hay (3.5 kg). This is supplemented with substantial feed in the form of crushed barley (2 kg), triticale (2 kg), and rapeseed (3 kg), as well as protein concentrate (1 kg) and a mineral and vitamin mix (0.2 kg). In the spring–summer season (from May to September), the main feed is pasture. The land for grazing consists of 80% of fodder grass mixtures—perennial ryegrass (*Lolium perenne* L.), cock’s-foot (*Dactylis glomerata* L.), common meadowgrass (*Poa pratensis* L.), meadow fescue (*Festuca pratensis* Huds.), and meadow cat’s-tail (*Phleum pratense* L.)— and 20% legumes, such as white clover (*Trifolium repens* L.), red clover (*Trifolium pratense* L.), and black medick (*Medicago lupulina* L.), and herbs like ribwort plantain (*Plantago lanceolata* L.), yarrow (*Achillea millefolium* L.), and dandelions (*Taraxacum officinale* Web.). The rest of the ration consists of fodder in the form of hay silage (10 kg), maize silage (10 kg), and meadow hay (3 kg); this is supplemented with energy feed (6 kg), dry beet pulp (1 kg), and buffering substances (0.2 kg).

In summer, the cows graze on meadows and pastures located 1.5 km from the barn in a plot system, with constant access to water. After the evening milking in the barn, the animals spend the night in the cattle yard, where they are given hay at will. During the winter, the animals remain in the barn around the clock. The litter is straw, which the cows eat ad libitum.

### 2.3. Animals and Milk Collections

This study made use of four first-calf heifers and sixteen older cows of the Polish Holstein–Friesian breed (up to the seventh lactation) in the barn. The average age of the herd was 2.7 lactations, the average body weight was 700 kg, and the cows scored 3.5–4 on the Body Condition Scores (BCS) scale. The average daily milk yield of the herd in the spring and summer was 31.8 liters of milk, while in the autumn and winter this was 6.8 liters higher. Milking took place twice a day between 6:30 and 17:30 using a milking machine (Alfa Laval AB, Lund, Sweden). The morning milk was always used to produce cheese using traditional methods. A representative sample of 100 mL was taken for laboratory analysis from each batch of milk intended for cheese production.

The tests were carried out on milk collected individually from 20 cows on the day of making the cheese and on soft rennet cheese made from the bulk milk in the two production seasons of summer (July–September) and winter (January–March). A total of 24 cheeses were made at two-week intervals in individual months, twelve from whole milk and twelve from skimmed milk.

### 2.4. Soft Cheese Making

Five liters of milk were pasteurized at 75 °C for about 25 s (FJ 15 Eco mini pasteurisator, Milky, Althofen, Austria) and then cooled to about 35 °C, before being inoculated with heterofermentative MSE (Motor Sich, Zaporizhia, Ukraine) (*Lactococcus lactis* subsp. lactis, *Lactococcus lactis* subsp. cremoris, *Lactococcus lactis* subsp. *Lactis biovar* diacetylactis, *Leuconostoc*, *Mesenteroides* subsp. cremoris). After 30 min of bacterial culture, dilute calcium chloride was added, according to the manufacturer’s instructions. Next, natural liquid rennet at 1:18000 (2 drops per liter milk) was added to form a curd. After 60 min, the curd was cut into large 1.5 cm cubes, and then into smaller cubes. After being cut, the curd was allowed to settle for 2–3 min. Subsequently, it was gently heated to 36 °C and then left for about 10 min. Once the cheese grain had clearly separated from the whey, the whey was drained, and the cheese slurry was transferred to a 20 cm diameter mold for drainage. After cooling, the cheese was cut into 250 g pieces, packed in plastic bags, and refrigerated at 4 °C for transport to the laboratory.

Cheese was made from skimmed milk in a similar manner. The skimmed milk was produced by centrifuging the cream from the whole milk using a Motor Sich 100-19 separator. The milk fat was measured using the Gerber method [16]. After centrifuging, the milk contained 0.5% fat.

The cheese made from the whole milk had an average moisture content of 67.3%, while that made from skimmed milk had a moisture level of 62.2%, according to Association of Oficial Analytical Chemists (AOAC) International [17].

The cheese yield (kg) was determined after 20–22h of storage at 4 °C. Yield (%) was calculated as the weight of cheese divided by the weight of the milk used. All cheeses were produced in accordance with local guidelines on good manufacturing practice and good hygienic practice.

### 2.5. Physicochemical Analysis

The milk was analyzed for pH, basic composition, somatic cell count, and color. The pH was measured at room temperature using Handylab 2 pH meter apparatus (Schott Geräte, Mainz, Germany) with a glass–calomel electrode (Schott L68880, Mainz, Germany). Standard buffers (Chempur, Piekary Sląskie, Poland) (pH 4.01; 7.00) were used for calibration.

The basic composition of the milk (dry matter, fat, protein, casein, lactose) was determined by a Milkoscan FT 120 instrument (Foss Electric, Hillerod, Denmark).

Somatic cell count (SCC) was evaluated using a Bacto Count IBCm (Bentley, MN, USA). The instrument applies laser-based flow cytometry, with ethidium bromide used to stain DNA in somatic cells. The laser-based counting section uses the fluorescence characteristics of the dye to count the cells one by one.

Milk color was determined using a Minolta CR-5 colorimeter (Minolta, Osaka, Japan) in terms of the CIE L*a*b* system, where L* represents lightness and ranges from 0 for black to 100 for white, a* represents the color’s position between green (−a*) and red (+a*), and b* represents the color’s position between blue (−b*) and yellow (+b*).

The cheese was analyzed for pH, titratable acidity, texture (firmness and stickiness), color, and organoleptic assessment. The pH and cheese color were determined as in the case of the milk.

Titratable acidity analysis was carried out using the Soxhlet–Henkel method (SH) and was determined using 0.25 N NaOH and phenolphthalein as an indicator [18].

### 2.6. Rheological and Profile Texture Analysis

Cheese texture analysis (firmness and stickiness) was performed using a TA-TX Plus micro stable texture analyzer (Micro Stable Micro Systems, Golborne, Warrington, UK). One-centimeter cubes of cheese were used. The cheese samples were conditioned at a measuring temperature of 18 °C for at least 1 h before testing. The samples were tested with a 0.5-in-diameter spherical probe, which was submerged at a speed of 1.5 mm/s to a depth of 10 mm before being returned to the starting position. Eight measurements were made on each cheese; the results were analyzed using the FarPoint Technologies program (Morrisville, NC, USA).

### 2.7. Organoleptic Analysis

The cheeses were organoleptically assessed for color, taste, smell, texture, and general appearance by a trained five-person panel (females, 35–50 years old) on a five-point scale (1: liked least; 5: liked most). Cheese blocks were cut into standard, bite-sized pieces, with each piece measuring 1.3 × 0.9 × 0.9 cm [19]. The samples were presented to the evaluators in a random order and marked with a 3-digit code. Some water was provided between each sample.

### 2.8. Statistical Analysis

SAS statistical package v 9.4 (SAS Institute Inc., Cary, NC, USA) was used for statistical analysis (2019). The SCC in the milk was subjected to logarithmic transformation before statistical verification [20].

The composition of whole milk in summer and winter season was compared with Student’s t-test.

In order to determine the significance of the experimental factors (season and type of milk) and the quality of the milk, physicochemical, rheological and organoleptic traits of cheese, a two-way analysis of variance was performed (GLM-SAS procedure v. 9.4 2019, SAS Institute Inc., Cary, NC, USA) according to the following linear model:

y_ijk_ = µ + s_i_ + c_j_ + (sc)_ij_ + e_ijk_, where y_ijk_ is the phenotypic value of the trait; µ is the overall mean, s_i_ is the fixed effect of the i-th season of milk collection (i = 1, 2); c_j_ is the fixed effect of the j-th type of milk (j = 1, 2); (sc)_ij_ is s x c interaction; eijk is the random residual effect. A detailed comparison of object means was made using Tukey’s test. Differences were considered significant at *p* < 0.05.

## 3. Results and Discussion

### 3.1. Milk Composition

The chemical composition of raw milk and the somatic cell counts (SCCs) are shown in Table 1. The content of basic milk components was typical of Holstein–Friesian cows [21]. In the winter season, higher total dry matter content was found, including that of fat and protein, than in the summer season: there was 11% more fat and 9% more protein. However, no significant changes were found in the lactose content.

Bernabucci et al. [22] obtained similar results. The differences in the basic composition of milk from different seasons were mainly associated with the composition of the feed [23]. It is well known that the composition of raw milk determines the nutritional and biological properties of products made from it and affects the yield of the cheese production process [24,25].

The average number of milk somatic cells found was 239,000 per mL (Table 1), which is within the normal range of below 400,000 per mL (Regulation EC No. 853/2004) [26]; this did not show significant seasonal differences. We noted only an increasing trend in the winter milk, associated with the cows’ calving period; this was also confirmed by Kul et al. [27]. In addition, the increased SCC content of the milk during the winter, when the cows were in the barn, could be associated with a lower level of herd hygiene, which usually leads to increased susceptibility to mastitis. In contrast, Alhussien and Dang [28] report higher SCC in summer milk. They suggest that high temperatures do not have an effect on the animals’ stress levels, but reduce the quality of feed consumed. A lack of nutrients in pasture fodder can reduce resistance in the animals, and consequently lead to an increase in bacterial infections. It should also be mentioned that the increase in SCC in milk leads to modifications in its chemical composition. This is important in cheese production, where it manifests as a decrease in casein content—the basic milk protein, which is of great importance from a technical point of view. In addition, levels of fat, lactose, and calcium ions are also reduced [29]. All these changes reduce the yield of the cheese-making process [30]. In summer, and especially in hot weather, the coagulation properties of milk deteriorate, resulting in changes in the protein fraction of casein, and especially in αS-CN and β-CN [22]. The extended rennet coagulation time may lead to improper formation or even a lack of curd, and thus disturb the production schedule [31]. Therefore, [31] they propose dividing milk into “well coagulating” and “poorly coagulating” categories, which will determine its suitable “best use”.

### 3.2. The Physicochemical Analysis of the Milk

Our physicochemical analysis of the milk by season, fat content and season × type of milk interaction is presented in Table 2. The influence of season, type of milk and interactions on all analyzed parameters was found. The obtained pH values of the milk prove the freshness of the analyzed raw material and, at the same time, its good suitability for processing. This parameter is one of the factors influencing milk coagulation; at a lowered pH, the milk coagulation process is faster. The pH of the whole milk ranged from 6.54–6.60, and was lower in summer than in winter (*p* < 0.05). However, Brodziak et al. [32] found that the milk of Polish Holstein–Friesians tends to have a higher pH (6.73–6.75) and not to show the impact of the production season. In skimmed milk, a significant increase in the pH value was found in relation to whole milk, while in the studies by Madadlou et al. [19] no statistical differences were found. The differences observed in all the milk color parameters in our own research related mainly to the b* (yellowness) parameter. This had the highest value in whole milk from the summer season, and the lowest in skimmed milk in the winter season (*p* < 0.05).

The high value of b* in the milk of cows grazing on pastures is associated with the presence of β-carotene in the feed [23,33]. According to Winkelman et al. [34], as animals graze, the sward changes, resulting in a significantly lower β-carotene concentration in milk fat in the summer than when the cows are on fresh pasture in spring. It should be noted that β-carotene is a potential biomarker for the milk of pasture-fed cows [35]. Generally, genetic (breed) and non-genetic (milking time, stage of lactation, parity) factors are closely related to milk color parameters (L*, a*, b*) [36].

### 3.3. Cheese Yield, the Physicochemical and Rheological Analysis of the Cheeses

Seasonal differences in the milk parameters were reflected in the physicochemical parameters of the cheese (Table 3). The effect of the season, type of milk and season x type of milk interaction on cheese yield, cheese acidity, color, and rheological parameters has been demonstrated. Cheese yield was not affected by the season, only by the type of milk; there were also no interactions. Cheeses made of whole milk had a higher average yield (20.58%) than cheeses of skimmed milk (16.28%), which is also confirmed by the studies of Madadlou et al. [19].

The acidity of the cheese, as measured by the pH, was higher in whole milk than in skimmed milk cheese (*p* = 0.0029). Statistically significant interactions between the season and the type of milk were found in the case of color (L, a*, b*) as well as firmness and stickiness. It was found that the type of milk used for production had a greater effect on the pH than the season did. Analysis of the titratable acidity of cheese confirmed the pH, with the highest acidity being found in the full cheese produced in summer, and the lowest acidity in the skimmed cheese made in winter. These differences could have been caused by the higher activity of the lactic acid bacteria inoculated during production, as they had a more favorable temperature and milk composition for reproduction and development in the summer whole milk than in the winter skimmed milk. Instrumental analysis of cheese color showed lower lightness (L*), greater green (−a*), and less yellow (b*) in the skimmed milk cheeses than in the whole milk cheeses (*p* = 0.001). The cheese-making season only had an impact on b*; cheeses made from summer milk were more yellow than those made from winter milk (*p* = 0.0001).

The color of dairy products depends mainly on the concentration of carotenoids [37]. It should be emphasized that the falsification of cheese is quite common in practice, with goat’s milk often being replaced by the cheaper cow’s milk. Spectrophotometric analysis is a valuable diagnostic tool in this regard [38].

The cheeses made from skimmed milk were harder and more elastic than the whole milk cheeses, regardless of the season (Table 3). As many authors have shown, the rheological properties of cheeses depend mainly on their basic composition, and above all on their protein and water contents [19,39,40]. Removing the fat from milk increases the proportions of these ingredients, making the cheese harder [41]. Milk fat usually ensures smoothness in full-fat cheeses by evenly distributing the casein fraction of the cheese; after its removal, casein plays the most important role in shaping the texture of the cheese. In addition, many other factors, such as milking time, the parameters of milk heat treatment, additives, and storage time of the cheese, affect the texture [42,43,44].

### 3.4. Organoleptic Analysis of the Cheeses

**Figure 1:**
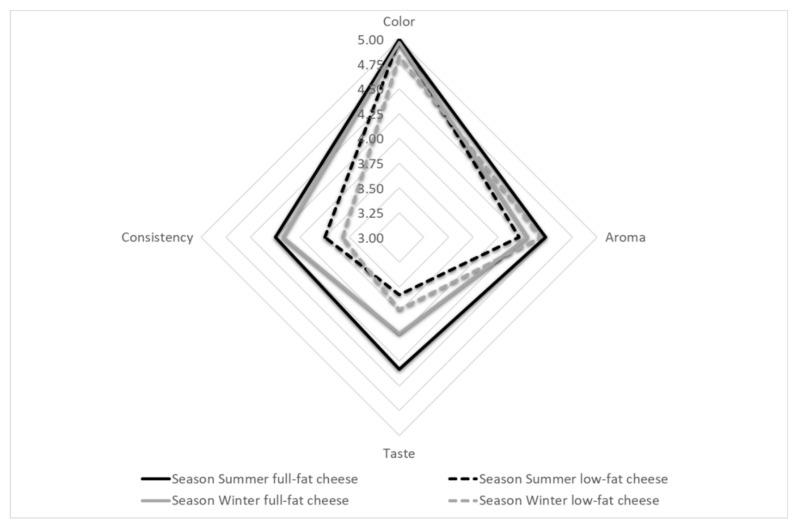
Organoleptic assessment of the cheeses (points).

An organoleptic assessment of the cheeses (Table 4; Figure 1) did not show any effect of season on individual parameters, other than color. Only the type of milk used for production differentiated all the parameters (with the exception of aroma and color). The cheeses were of uniform colors, assessed as cream-yellow in the case of whole milk, and white in the case of skimmed milk. The statistically significant interaction of season x type of milk allows for the deterioration of the taste of whole milk cheese in winter compared to summer, while the opposite relationship was observed in the case of skimmed milk cheese. The cheeses scored very highly, although the cheese from winter skimmed milk was rated slightly better than the summer skimmed milk cheese (*p* = 0.0001).

There were no differences in cheese aroma (*p* > 0.05). In contrast, Bergamaschi and Bittante [45] reported a very broad spectrum of fragrance compounds in dairy products from cows that grazed on Alpine pastures. Alcohols, aldehydes, esters, free fatty acids, ketones, lactones, sulfurs, terpenes, phenols, and benzenes were among the compounds identified in them. Therefore, in order to better understand the sensory attributes of low-fat cheese produced in traditional family farms, further research on the profile of volatile compounds is needed.

The whole milk cheeses were rated most favorably in terms of taste, while the taste of the skimmed milk cheeses was less pronounced. A less pronounced taste is usually found in cheeses with a lower fat content than in full-fat cheeses. This might be due to the taste being diluted as a result of excessive moisture retention [46]. Amelia et al. [47] found that when the fat content of Cheddar cheese decreased, the intensity of sweet, sour, and umami tastes significantly decreased; additionally, a bitter taste was noted in low-fat cheddar. Absent or barely perceptible taste are usually counted among cheese’s most undesirable taste characteristics [41]. In addition, an important factor that affects the sensory characteristics of cheeses is the geographical area in which the animals are grazed, and the associated botanical composition of the pasture sward [23]. Rich flavors are characteristic of cheeses made from the milk of cows grazing on mountain pastures. In the study by Bugaud et al. [48], such cheeses were more often rated as “fruity”, “animal”, “boiled milk”, or “hazelnut”, and less pungent than cheeses made from milk produced from valley pastures. It should be noted that a cheese’s taste and aroma are shaped during ripening by the transformation of carbohydrates, proteins and fats.

**Figure 2:**
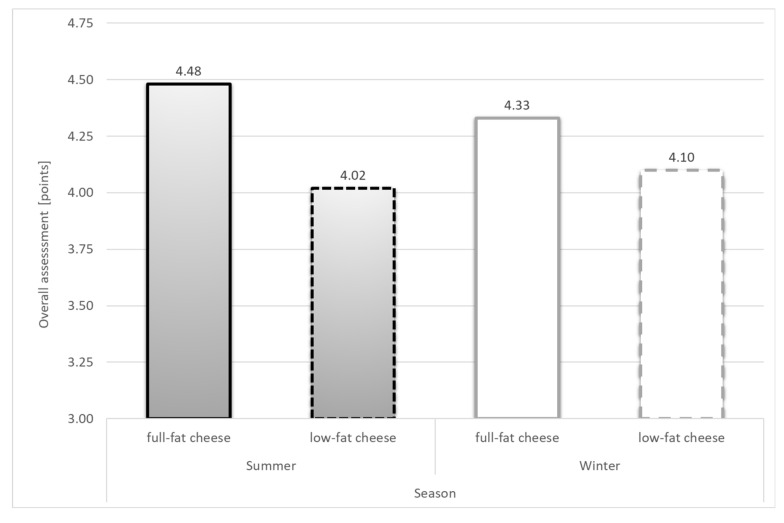
Overall assessment of the cheeses (points).

In terms of consistency, the highest rated ones in our study were the full-fat cheeses from the summer season, while the low-fat cheeses from winter were rated lowest (*p* ≤ 0.05). The consistency of the whole milk cheeses was assessed as homogeneous, compact, crumbly, and with small holes; the skimmed cheeses were described as hard, lumpy, rubbery, and difficult to chew. To the surprise of the evaluators, cheeses made from skimmed milk received a satisfactory overall assessment, though, as expected, this was significantly lower than that of whole milk cheeses (Table 4; Figure 2).

The analysis shows that the total removal of fat from milk significantly reduced the taste, texture, and overall evaluation of cheeses, as has been confirmed in many studies [19,49]. Research is still underway to improve the taste of skimmed milk cheese, where the careful selection of starter bacteria is likely to play an important role [50].

## 4. Conclusions

Data show that the milk of these Polish Holstein–Friesian cows has the correct chemical composition and good cytological quality. The production of soft, unripe rennet cheese from this milk is a good alternative for medium-sized traditional farms looking for additional income. We found that both the season and the fat content of the milk affected the physicochemical and rheological parameters of the cheese produced from it. The fat content of the milk had a greater effect on the organoleptic assessment of the cheese. The skimmed milk cheeses, despite their greater firmness and stickiness, received satisfactory organoleptic assessments, indicating that they can serve as replacements for full-fat cheeses for people looking for low-fat products.

## Figures and Tables

**Table 1:** Chemical composition of milk and somatic cells count depending on the season.

Item	Season						*p*-Value
	Summer			Winter			
	*x*	SD	Range	*x*	SD	Range	
Dry matter (%)	12.30	0.78	10.83–14.59	13.06	1.17	11.15–16.32	0.0003
Fat (%)	3.75	0.67	2.07–5.95	4.15	0.85	2.85–7.10	0.0115
Protein (%)	3.20	0.35	2.65–4.19	3.49	0.60	2.79–5.79	0.0041
Casein (%)	2.49	0.27	2.06–3.29	2.72	0.46	2.11–4.48	0.0042
Lactose (%)	4.66	0.20	4.32–5.22	4.69	0.44	3.02–5.12	0.6432
SCC (×10^3^ /mL)	232	161	8–400	246	149	5–400	0.6624

SCC—somatic cell count; *x*—arithmetic mean; SD—standard deviation.

**Table 2:** Physicochemical parameters of milk depending on the season and the type of milk.

Item		Season								Season	Type of Milk	Season × Type of Milk
		Summer				Winter				*p*-Value		
		Full-Fat Milk		Low-Fat Milk		Full-Fat Milk		Low-Fat Milk				
		*x*	SD	*x*	SD	*x*	SD	*x*	SD			
pH		6.54 ^a^	0.08	6.83 ^b^	0.02	6.60 ^c^	0.06	6.68 ^d^	0.03	0.0039	0.0001	0.0001
Color	L*	88.58 ^a^	0.24	86.65 ^b^	0.92	88.81 ^a^	0.39	88.48 ^a^	0.50	0.0001	0.0001	0.0001
	a*	−3.38 ^a^	0.32	−6.14 ^b^	0.47	−3.44 ^a^	0.10	−5.50 ^c^	0.23	0.0082	0.0001	0.0003
	b*	14.03 ^a^	0.87	9.19 ^b^	0.97	10.53 ^c^	0.44	7.58 ^d^	1.40	0.0001	0.0001	0.0018

L*—lightness; a*—redness; b*—yellowness; ^a–d^ Means within a row without a common superscript differ (*p* < 0.05); *x*—arithmetic mean; SD—standard deviation.

**Table 3:** Physicochemical and rheological analysis of the cheeses depending on the season and the type of milk.

Item		Season				SE	Season	Type of Milk	Season × Type of Milk
		Summer		Winter					
		Full-Fat Cheese *x*	Low-Fat Cheese *x*	Full-Fat Cheese *x*	Low-Fat Cheese *x*		*p*-Value		
Cheese yield [kg]		1.04	0.80	1.08	0.88	0.03	0.2062	0.0001	0.5817
pH		5.76	6.05	5.94	6.29	0.06	0.0493	0.0029	0.7444
SH		7.40	5.97	5.60	4.50	0.31	0.0074	0.0360	0.7792
Color	L*	91.25 ^a^	90.81 ^b^	91.71 ^a^	90.60 ^b^	0.09	0.3862	0.0001	0.0220
	a*	−1.60 ^a^	−2.80 ^b^	−1.34 ^a^	−2.94 ^b^	0.09	0.4564	0.0001	0.0090
	b*	18.11 ^a^	13.31 ^b^	15.24 ^c^	12.96 ^b^	0.26	0.0001	0.0001	0.0001
Firmness (g)		566.78 ^a^	833.47 ^b^	548.98 ^a^	1137.82 ^c^	39.52	0.0378	0.0001	0.0198
Stickiness (g)		−10.66 ^a^	−4.04 ^b^	−3.49 ^b^	−1.02 ^c^	0.51	0.0001	0.0001	0.0117

SH—titratable acidity; L*—lightness; a*—redness; b*—yellowness; ^a–c^ means within a row without a common superscript differ (*p* < 0.05); *x*—arithmetic mean; SE—standard error of mean.

**Table 4:** Organoleptic assessment of the cheeses depending on the season and the type of milk.

Item	Season				SE	Season	Type of Milk	Season × Type of Milk
	Summer		Winter					
	Full-Fat Cheese *x*	Low-Fat Cheese *x*	Full-Fat Cheese *x*	Low-Fat Cheese *x*		*p*-Value		
Color	5.00	5.00	4.97	4.83	0.02	0.0053	0.0607	0.0607
Aroma	4.48	4.21	4.30	4.43	0.05	0.8064	0.4961	0.0577
Taste	4.33 ^a^	3.58 ^b^	3.97 ^a^	3.73 ^a–b^	0.06	0.3141	0.0001	0.0174
Consistency	4.25	3.75	4.17	3.57	0.06	0.2151	0.0001	0.6412
Overall assessment	4.48	4.02	4.33	4.10	0.04	0.6162	0.0001	0.0923

^a–b^ Means within a row without a common superscript differ (*p* < 0.05); *x*—arithmetic mean; SE—standard error of mean.
